# Correction: BICC1 drives pancreatic cancer progression by inducing VEGF-independent angiogenesis

**DOI:** 10.1038/s41392-023-01721-z

**Published:** 2024-01-05

**Authors:** Chongbiao Huang, Hui Li, Yang Xu, Chao Xu, Huizhi Sun, Zengxun Li, Yi Ge, Hongwei Wang, Tiansuo Zhao, Song Gao, Xiuchao Wang, Shengyu Yang, Peiqing Sun, Zhe Liu, Jing Liu, Antao Chang, Jihui Hao

**Affiliations:** 1 Department of Pancreatic Cancer, Tianjin Medical University Cancer Institute and Hospital, National Clinical Research Center for Cancer, Tianjin Key Laboratory of Digestive Cancer, Key Laboratory of Cancer Prevention and Therapy, 300060 Tianjin, China; 2 Department of Anorectal Surgery, The Second Hospital of Tianjin Medical University, Tianjin, China; 3 Department of Cellular and Molecular Physiology, the Pennsylvania State University College of Medicine, Hershey, PA USA; 4 Department of Cancer Biology, Wake Forest Baptist Comprehensive Cancer Center, Wake Forest Baptist Medical Center, Winston-Salem, NC USA; 5 Department of Immunology, School of Basic Medical Sciences, Tianjin Medical University, Tianjin, China

**Keywords:** Tumour angiogenesis, Gastrointestinal cancer

Correction to: *Signal Transduction and Targeted Therapy* 10.1038/s41392-023-01478-5, published online 14 July 2023

After online publication of the article, the authors noticed one inadvertent mistake in Fig. 4f. The image of BICC1 IHC in the BICC1+LCN2 ab group was repeatedly inserted as the image of BICC1+CXCL1 ab group by mistake during the final revision process. The correct figure was provided as follows. The correction did not affect any of our results or discussion as present in the original publication. We apologize for this inadvertent mistake.
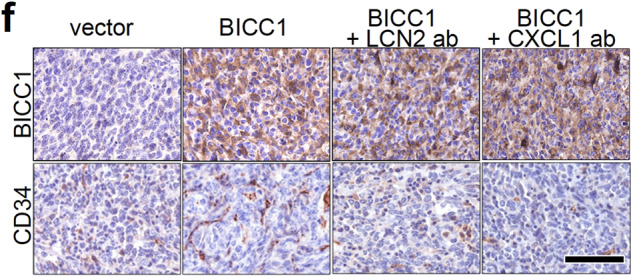


The original article has been corrected.

